# Effects of Gallic Acid and Cyclosporine A on Antioxidant Capacity and Cardiac Markers of Rat Isolated Heart After Ischemia/Reperfusion

**DOI:** 10.5812/ircmj.16424

**Published:** 2014-06-05

**Authors:** Mohammad Badavi, Najmeh Sadeghi, Mahin Dianat, Alireza Samarbafzadeh

**Affiliations:** 1 Physiology Research Centre, Department of Physiology, Faculty of Medicine, Ahvaz Jundishapur University of Medical Sciences, Ahvaz, IR Iran; 2 Atherosclerosis Research Center, Golestan Hospital, Ahvaz Jundishapur University of Medical Sciences, Ahvaz, IR Iran; 3 Diabetes Research Center, Department of Physiology, Faculty of Medicine, Ahvaz Jundishapur University of Medical Sciences, Ahvaz, IR Iran; 4 Infectious and Tropical Diseases Research Center, Department of Virology, Faculty of Medicine, Ahvaz Jundishapur University of Medical Sciences, Ahvaz, IR Iran

**Keywords:** Antioxidant Capacity, Cyclosporine A, Ischemia Reperfusion, Gallic Acid, Myocardial Infarction

## Abstract

**Background:**

Myocardial infarction is one of the important causes of death during old ages. Gallic acid as an antioxidant or cyclosporine A (CsA) as inhibitor of mitochondrial permeability transition pore (mPTP) alone could prevent these complications to some extent, but their combination effect has not been investigated.

**Objectives:**

The aim of this study was to determine the combined effect of gallic acid and CsA on antioxidant capacity of isolated heart tissues during ischemia reperfusion.

**Materials and Methods:**

Eighty male Wistar rats were randomly assigned to different groups: sham, control (Ca, received saline, 1 mL/kg); 3 groups were pretreated with gallic acid (G1a: 7.5, G2a: 15, G3a: 30 mg/kg) for 10 days, and the other 3 groups were pretreated with gallic acid and received CsA (0.2 µM) for 10 minutes before induction of ischemia and during the first 10 minutes of reperfusion (G1b, G2b and G3b) and the last group received CsA alone (Cb). After 10 days of pretreatment, the heart was isolated and transferred to the Langendorff apparatus and exposed to 30 minutes ischemia followed by 60 minutes of reperfusion. After that cardiac markers and antioxidant enzymes were assessed in cardiac tissues.

**Results:**

Lactate dehydrogenase (LDH), Superoxide dismutase (SOD), catalase (CAT) and glutathione peroxidase (GPX) activity increased and malondialdehyde (MDA) decreased in animals pretreated with gallic acid significantly. However, pretreatment with gallic acid followed by CsA during reperfusion improved the antioxidant capacity and cardiac marker enzymes and restored the lipid peroxidation more effective than gallic acid or CsA alone. Nevertheless, CsA did not change the cardiac marker enzymes significantly.

**Conclusions:**

Gallic acid and CsA combination improved antioxidant capacity and cell membrane integrity more than each one alone. Therefore, it can be a therapeutic approach to reduce the I/R injury.

## 1. Background

Ischemic heart disease (IHD) is a common disease in society and myocardial infarction has been one of the important causes of death during the recent years (1). It occurs because of deficient of oxygen in the myocardium due to insufficient of blood supply leading to free radical production. Free radicals may combine with phospholipids of cell membrane and make proxy radicals and lipid hydro peroxidation (2). Lipid peroxidation results in malondialdehyde (MDA) formation as a cytotoxic product via membrane disruption and finally leads to cell damage and necrosis. Then mitochondrial dysfunction due to fatty acid oxidation may develop and increase ischemia induced injury (3). It has been reported that mitochondrial reactive oxygen species (ROS) increase oxidative phosphorylation disorder and disturb the complex I and IV activity. These enzyme activities can be restored to normal levels by potentiating antioxidant defense system via inducing the antioxidant enzymes such as superoxide dismutase (Mn-SOD) and catalase (4). Previous studies have also shown that glutathione and thiol status are reduced during ischemia and concomitant decrease of SOD activity, but during reperfusion, oxidized glutathione is released. These defensive mechanisms are activated which protect the organism against oxidative stress (5, 6). In addition, during ischemia the myocardial cell membranes are damaged and some of enzymes such as lactate dehydrogenase (LDH) can be released. To overcome this condition and restore myocardial function, the main strategy is reperfusion of the ischemic tissue, but reperfusion could result in more free radical formation like reactive oxygen species (ROS) in mitochondria. Therefore, LDH, Creatine-phosphokinase-myocardial (CPK-MB) and creatine kinase (CK) can be released and may be used as markers to diagnose the myonecrosis level (7, 8). In recent years, some strategies have been proposed for cardioprotection against oxidative stress. They are using natural antioxidants by eating fresh vegetables and fruits. Because they have antioxidants such as flavonoids, anthocyanin and polyphenol compounds (9). Inducing imbalance between pro-oxidants (free radicals) and antioxidant capacity presented in cell in normal condition brings ROS production and opening of the mitochondrial permeability transition pore (mPTP). Opening of (mPTP) leads to increased ROS production (10) more and more and, a vicious cycle (11) is formed which worsens the oxidative stress. Therefore, preservation of the mitochondria by mPTP inhibitors can be effective. On the other hand, previous studies have reported that inhibition of mPTP by cyclosporine A (CsA) preserves mitochondrial morphology against oxidative stress derived I/R injury and limits the myonecrosis and apoptosis (12). In addition, other researchers have demonstrated that resveratrol (a polyphenol compound present in red wine) induced the thioredoxin (Trx-1), hemoxygenase (HO-1), and vascular epidermal growth factor (VEGF) via NO in diabetic myocardium and reduced the cardio myocyte death by increasing the SOD activity during I/R (13). Besides, other studies have shown that gallic acid (an endogenous plant phenol found in tea leaves, grape seeds and red wine and a metabolite of propyl gallate) improved antioxidant status and protected heart and lysosomal membrane against oxidative stress in isoproterenol induced cardio toxicity in Wistar rats (14). Mitochondria as key executors in cell respiratory can be more effective object for cell survival against I/R injury. According to previous studies, antioxidant effect of gallic acid has been investigated in cardio toxicity (14), but its potential effects in combination with CsA in isolated ischemic heart have not been investigated. Gallic acid can play a favorable role in scavenging free radicals, which improves the mitochondrial dysfunction and potentiates the antioxidant capacity.

## 2. Objectives

The aim of this study was to determine the effect of gallic acid as an antioxidant in combination with CsA as an mPTP inhibitor on antioxidant capacity in rat isolated ischemic heart.

## 3. Materials and Methods

### 3.1. Animals

Eighty male Wistar rats (250-300g) used in this experimental study were obtained from the animal house of Ahvaz Jundishapur University of Medical Sciences. The animals were randomly assigned into 9 groups (Table 1). The sham group were not exposed to ischemia, control group received normal saline as gallic acid solvent for 10 days then exposed to ischemia (Ca); also, CsA (Cb) was administrated to some of animals in this group . The Gallic acid was dissolved in normal saline and administered once a day orally (G1a: 7.5, G2a: 15, G3a: 30 mg/kg) via a gavage needle for 10 days (14). In some groups, the animals were firstly pretreated by gallic acid and then exposed to CsA administration (G1b, G2b and G3b). CsA was dissolved in ethanol, added to perfusion solution (0.2 µM) and administered ten minutes before induction of ischemia and during the first 10 minutes of reperfusion (15) (Table 1). All groups were housed under the same conditions (room temperature of 23 ± 2^°^C, with a 12-hour dark-light cycle, free access to food and water). All procedures were performed in accordance to the standards for animal care, approved by the ethical committee of Ahvaz Jundishapur University of Medical Sciences (AJUMS.REC.1392.222).

**Table 1.** Animals’ Groups (n = at Least 8, in Each Group) That Have Used in This Study

Sham ^+^^a^	Gallic Acid, mg/kg	Cyclosporine A, µM
**C1 **		
C_a_^b^	Saline	0
C_b_^c, d^	Saline	0.2
**G1^e^**		
G_1a_	7.5	0
G_1b_	7.5	0.2
**G2**		
G_2a_	15	0
G_2b_	15	0.2
**G3**		
G_3a_	30	0
G_3b_	30	0.2

^a^ + Positive control =a group that didn’t expose to ischemia.

^b^ a = Groups that did not received CsA.

^c^ b = Groups that received gallic acid + CsA.

^d^ C = Control groups.

^e^ G = Groups that received gallic acid.

Drugs: gallic acid, CsA and heparin were purchased from Sigma (St. Louis, MO USA), sodium chloride, potassium chloride, magnesium sulphate, sodium hydrogen carbonate, potassium hydrogen orthophosphate, D-glucose and calcium chloride were obtained from Merck Laboratories, and Ketamine and Xylazine from Alfasan Co. (Woerden-Holland).

### 3.2. Preparation and Isolated Heart Perfusion

The animals were anesthetized using Ketamine HCl (50 mg/kg) and xylazine (5 mg/kg). Heparin (1000 U/kg, IP) was administrated 20 minutes before the operation. The trachea was cannulated and the animals were ventilated with room air using a rodent ventilator (UGO BASILE, model: 7025) (16). Chest was opened via thoracotomy and removing the ribs, and then artificial respiration started immediately (with a tidal volume of 1.5 mL/100 g body weight and rate of 70) for maintaining PCO_2_, PO_2_ and pH parameters in normal range. After that pericardium was raptured, aorta was exposed and a steel cannula was placed into the aorta and secured with a suture. The hearts were immediately perfused with Krebs-Henseleit bicarbonate buffer at a constant pressure of (60-70 mmHg) and temperature of 37°C. Buffer was bubbled with 95% O_2_-5% CO2 to attain a pH of 7.4, 20 minutes before the operation. The heart was quickly excised and transferred to a Langendorff apparatus while continuously perfused (16). All hearts were perfused for 25-30 minutes before the induction of ischemia to attain the equilibrated Coronary Perfusion Pressure (CPP), and then subjected to 30 minutes of no flow global ischemia, followed by 60 minutes of reperfusion (17). Hearts that showed arrhythmia during the equilibration time or sustained reduction in CPP or flow less than 8-9 mL/minutes were excluded from the study. ECG, CPP and perfusion flow were monitored before induction of ischemia and during reperfusion using the Power lab recorder (ADInstruments, Australia). Successful induction of ischemia was determined by ST elevation on electrocardiogram.

### 3.3. Assay of Cardiac Marker Enzymes

Myocardial injury was measured by lactate dehydrogenase (LDH) efflux. Perfusate from isolated perfused heart was collected at 5, 10, and 15 minutes after reperfusion. LDH, creatine kinase (CK) and myocardial creatine kinase (CK-MB) were assayed spectrophotometrically, using standard commercial kits from Sigma-Aldrich (USA) and were expressed as logarithm of units per liter (17).

### 3.4. Antioxidant Enzyme Activity of Heart

Antioxidant capacity was measured in heart tissues. Five hundred milligrams of heart tissue was homogenized using homogenizer (Heidolph Silent Crusher M, Germany) in 5 mL PBS (50 mM, pH = 7.4) and centrifuged at 4000 rpm for 10 minutes. Then the supernatant was collected and stored at -80^°^C for assay. The amount of protein in supernatant was assessed by total protein kit (Pars Azmun Co) (18). Superoxide dismutase (SOD), glutathione peroxidase and total antioxidant activities were measured by Randox kits (Randox Lab, UK). A catalase (CAT) activity was investigated at 37°C according to Aebi method (19). One unit of CAT activity was described as 1 µmol of H_2_O_2_ per minute at 37°C, which was degraded by hydrogen peroxidase at 240 nm. Specific activity was determined by converting H_2_O_2_ in µmol/minute mg protein to U/Mg protein in the tissue (19).

### 3.5. Thiobarbituric Acid Reactive Substances (TBARS) Assay

TBARS was assayed as a marker of lipid peroxidation using the Buege method (20). Briefly, 250 µL homogenate was added to 2 mL reaction solution containing (Trichloroacetic acid 15%, thiobarbituric acid 0.37%, NHCl 0.25, 1:1:1, w/v) and heated at 100°C for 15 minutes. After cooling the mixture in room temperature, centrifuged (10000 g for 10 minutes) and the absorbance of supernatant was recorded at 532 nm using spectrophotometer (Unico UV-2100 spectrophotometer). The result was expressed as nmol per Mg protein in the homogenate (20).

### 3.6. Statistical Analysis

Results were analyzed using SPSS version 16 and expressed as Mean ± SEM. The data normal distribution was checked using the Kolmogorov-Smirnov goodness-of-fit test. Comparisons between groups were performed using repeated measurement ANOVA or one way ANOVA followed by LSD multiple comparison tests. P-values of less than 0.05 were considered significant.

## 4. Results

### 4.1. Activity of Cardiac Marker Enzymes

Cardiac marker enzymes activity as an index of myocardial cellular injury was measured after I/R. The data showed that LDH, CPK and CK-MB activity were increased after I/R compared to sham (LDH: 3 ± 0.5 vs. 1.9 ± 0.2, CPK: 1.5 ± 0.3 vs.0.7 ± 0 and CK-MB: 2.2 ± 0.3 vs. 1 ± 0.2 unit/mL). The time course of LDH in coronary effluent during reperfusion is shown in Table 2. LDH activity significantly increased in early reperfusion and peaked at 5 minutes after reperfusion. LDH activity in heart obtained from the group that received both gallic acid (7.5 mg/kg) and CsA (0.2 μM) was significantly higher than control, sham and CsA at first 15-minutes of reperfusion (G7.5 + CsA: 4.4 ± 0.6 vs. control: 3.0 ± 0.5, sham: 1.9 ± 0.2 and CsA: 1.5 ± 0.3 unit/mL, P ≤ 0.01, Repeated measurement ANOVA,). However, LDH activity was not significantly different between CsA and control or sham groups (at 5 minutes: 1.5 ± 0.3 vs. control: 3.0 ± 0.5, sham: 1.9 ± 0.2 unit/mL). Additionally, combination of gallic acid and CsA decreased LDH activity more than pretreatment with gallic acid alone; this reduction was significant in G15 + CsA compared with either G15 or control groups (at 5 minutes: G15 + CsA: 2.3 ± 0.3 vs. G15: 4.2 ± 0.5 and control: 2.66 ± 0.3, unit/mL, repeated measurement ANOVA P < 0.05, Mean ± SEM), (Table 2). CPK activity increased significantly in gallic acid (7.5 mg/kg) + CsA group compared with sham, CsA and gallic acid alone (7.5 mg/kg) groups (at 5 minutes: G 7.5 + CsA: 3.4 ± 1 vs. CsA: 1 ± 0.3, G 7.5: 1.8 ± 0.3, sham: 0.7 ± 0.0001, unit/mL, repeated measurement ANOVA , P < 0.01), (Table 2). Nevertheless, CsA alone did not affect CPK activity significantly. On the other hand, gallic acid (15 mg/kg) combined with CsA decreased CPK activity more significantly compared to control group (5 minutes: G 15 + CsA: 0.9 ± 0.1 vs. control: 1.5 ± 0.3, unit/mL, repeated measurement ANOVA, P < 0.05), (Table 2). CK-MB activity significantly increased in the presence of gallic acid (7.5 mg/kg + CsA) compared with control, sham, CsA and gallic acid alone (G 7.5 + CsA: 3.6 ± 0.3 vs. control: 2 ± 0.3, CsA: 1.4 ± 0.2, G 7.5: 0.9 ± 0.1, sham: 1 ± 0.2, unit/mL P ≤ 0.001), (Table 2). There was no significant difference in the presence of CsA and gallic acid (15 mg/kg + CsA), in CK-MB activity.

**Table 2.** Effect of Gallic Acid (G7.5, G15 and G30 mg/kg) and Combination of Gallic Acid With CsA (G7.5, G15 and G30 mg/kg + CsA) on LDH,CPK and CK-MB activity in Coronary Effluent After 30 min Ischemia Followed 60 min Reperfusion (Mean ± SD, n= at Least 8). Cyclosporine A (0.2 µM) Administered at the Onset of Reperfusion (CsA).^a^

Groups	Time		
	5	10	15
**LDH**			
Sham	1.89 ± 0.73	1.75 ± 0.92	1.75 ± 0.92
Control	2.65 ± 0.86	1.93 ± 0.71	3.03 ± 1.32 ^a^
G 7.5	3.28 ± 0.60	1.89 ± 1.33	2.74 ± 0.70
G 15	4.17 ± 1.12	2.80 ± 1.31	2.15 ± 1.31
G 30	2.67 ± 1.39	2.47 ± 0.90	2.94 ± 0.71
CsA	1.56 ± 0.67	2.79 ± 1.34	1.89 ± 0.98
G 7.5 + CsA	3.13 ± 0.89	3.79 ± 1.23	4.44 ± 1.46
G 15 + CsA	2.28 ± 0.73	1.95 ± 0.81	1.33 ± 1.33 ^b^
G 30 + CsA	2.23 ± 0.86	2.23 ± 1.13	2.38 ± 1.27
**CRP**			
Sham	0.69 ± 0.00	0.69 ± 0.00	0.69 ± 0.00
Control	1.48 ± 0.85	1.86 ± 1.05	1.62 ± 1.29 ^a^
G 7.5	1.82 ± 0.80	1.08 ± 0.65	1.02 ± 0.43
G 15	2.06 ± 1.38	1.51 ± 1.56	1.85 ± 1.44
G 30	1.33 ± 0.88	1.01 ± 0.60	1.11 ± 0.59
CsA	1.02 ± 0.72	1.84 ± 1.18	0.94 ± 0.35
G 7.5 + CsA	2.01 ± 0.59	2.79 ± 1.65	3.39 ± 1.97
G 15 + CsA	0.86 ± 0.22	0.97 ± 0.27	0.99 ± 0.30 ^b^
G 30 + CsA	1.15 ± 0.82	1.21 ± 0.55	1.07 ± 0.29
**CK-MB**			
Sham	1.01 ± 0.55	1.01 ± 0.55	1.01 ± 0.55
Control	1.36 ± 0.63	1.79 ± 0.71	2.21 ± 0.68 ^a^
G 7.5	1.93 ± 0.51	0.94 ± 0.29	1.42 ± 0.65
G 15	2.46 ± 1.03	2.50 ± 1.42	1.97 ± 1.15
G 30	1.38 ± 0.62	1.48 ± 0.24	1.29 ± 0.37
CsA	1.40 ± 0.56	1.46 ± 0.64	1.37 ± 0.48
G 7.5 + CsA	2.19 ± 0.36	3.24 ± 1.01	3.60 ± 0.67
G 15 + CsA	1.68 ± 0.22	1.16 ± 0.45	0.88 ± 0.41
G 30 + CsA	1.42 ± 0.42	1.36 ± 0.45	1.29 ± 0.60

^a^ Indicates significant difference with sham group (P < 0.05, repeated measurement ANOVA followed by LSD test).

^b^ Indicates significant difference with control group, P < 0.05.

### 4.2. Antioxidant Enzyme Activity and TBARS Level

**Figure 1.**
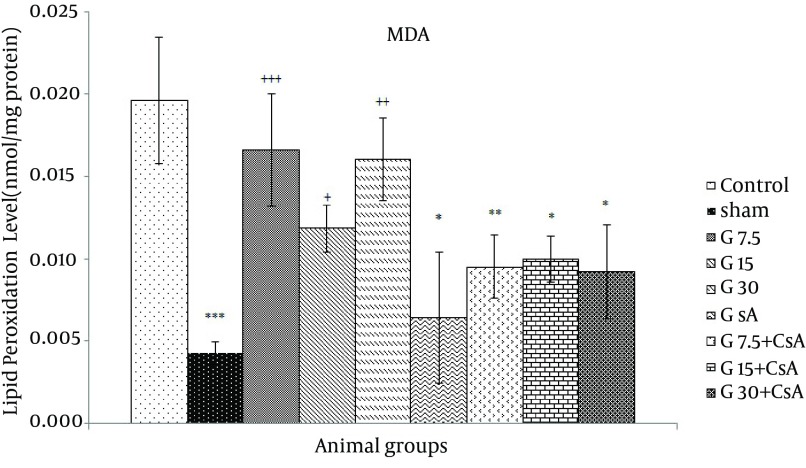
TBARS levels in Heart of Rats After 30 Minutes Ischemia and 60 Minutes Reperfusion (Mean ± SEM, n = at least 8) The animals received three different doses of gallic acid (G7.5, G15 and G30 mg/kg) for 10 days before the beginning of I/R studies. Cyclosporine A (0.2 µM) was administered at the onset of reperfusion (CsA). Control animals received normal saline as the solvent of gallic acid. Sham animals were not exposed to I/R. * indicates significant difference with control. + indicates significant difference with sham. (+ or * P < 0.05, ** or ++ P < 0.01, *** or +++, P < 0.001, one-way ANOVA followed by LSD test).

Lipid peroxidation increased significantly in the heart of control group exposed to I/R compared with sham group (0.02 ± 0.004 vs. 0.004 ± 0.001 nmol/mg proteins, respectively). TBARS level was reduced significantly in the presence of CsA compared with the control group (0.006 ± 0.004 vs. 0.02 ± 0.004, nmol/mg protein, P < 0.001), (Figure 1). Reduction of TBARS level in the heart received gallic acid alone was not significant, but combination of gallic acid (7.5, 15 and 30 mg/kg) and CsA was more effective than gallic acid alone (One-way ANOVA, followed by LSD, P < 0.05) (Figure 1).

**Figure 2.**
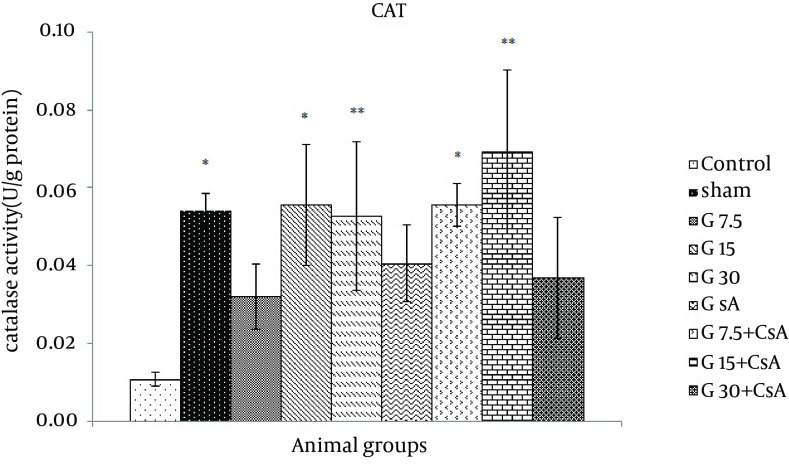
CAT Activity in Heart of Rats After 30 Minutes Ischemia and 60 Minutes of Reperfusion (Mean ± SEM, n = at least 8) The animals received three different doses of gallic acid (G7.5, G15 and G30 mg/kg) for 10 days before the beginning of I/R studies. Cyclosporine A (0.2 µM) was administered at the onset of reperfusion (CsA). Control animals received normal saline as the solvent of gallic acid. Sham animals were not exposed to I/R. * indicates significant difference with control. (* P < 0.05, ** P < 0.01, one-way ANOVA followed by LSD test).

A significant decrease in catalase activity was observed after I/R in control as compared to sham group (0.01 ± 0.002 vs. 0.047 ± 0.004, U/g protein). CAT level increased significantly in groups that received 15 or 30 mg/kg gallic acid alone and 7.5 or 15 mg/kg + CsA compared with control (0.05 ± 0.02, 0.06 ± 0.02, 0.06 ± 0.01, 0.07 ± 0.02 vs. 0.01 ± 0.002, U/g protein P < 0.05), (Figure 2).

**Figure 3.**
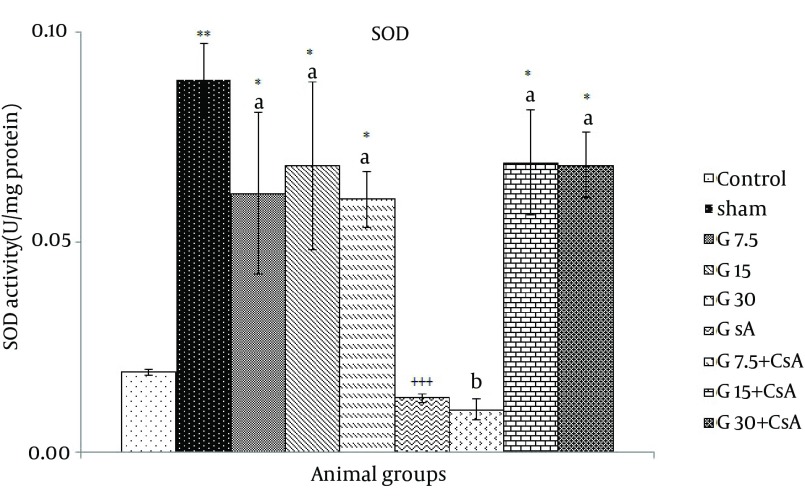
SOD activity in Heart of Rats After 30 Minutes Ischemia and 60 Minutes of Reperfusion (Mean ± SEM, n = at least 8) The animals received three different doses of gallic acid (G7.5, G15 and G30 mg/kg) for 10 days before the beginning of I/R studies. Animals received cyclosporine A (0.2 µM) at the onset of reperfusion (CsA); Control animals received normal saline as the solvent of gallic acid. Sham animals were not exposed to I/R.* indicates significant difference with control. + indicates significant difference with sham. (* or + P < 0.05, ** or ++ P < 0.01, +++ P < 0.001, one-way ANOVA followed by LSD test). a = compared with CsA, b = compared with each corresponding dose of gallic acid alone.

Superoxide dismutase activity was increased significantly in all groups except (G7.5 mg/kg + CsA) and CsA alone compared with control and CsA groups (G7.5: 0.06 ± 0.02, G15: 0.07 ± 0.02, and G30: 0.06 ± 0.01, G15 or G30 + CsA: 0.07 ± 0.01 vs. control: 0.02 ± 0.002, CsA: 0.01 ± 0.001 U/Mg protein, P ≤ 0.05), (Figure 3). SOD activity in a group that received 7.5 mg/kg gallic acid alone was increased significantly in comparison with gallic acid 7.5 mg/kg + CsA (0. 0.01 ± 0.001vs 0.06 ± 0.02 U/mg protein).

**Figure 4.**
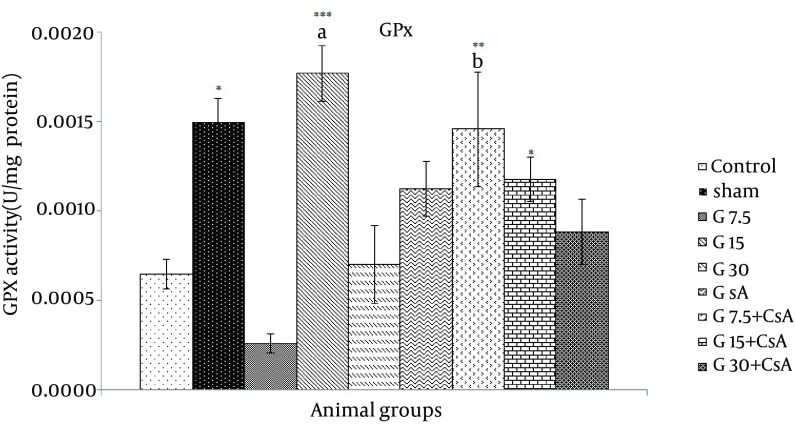
GPX Activity in Heart of Rats After 30 Minutes Ischemia and 60 Minutes of Reperfusion (Mean ± SEM, n = at least 8) The animals received three different doses of gallic acid (G7.5, G15 and G30 mg/kg) for 10 days before the beginning of I/R studies. Animals received cyclosporine A (0.2 µM) at the onset of reperfusion (CsA); Control animals received normal saline as the solvent of gallic acid. Sham animals were not exposed to I/R.* indicates significant difference with control.* indicates significant difference with sham. (P < 0.05, ** P < 0.01, *** P < 0.001, one-way ANOVA followed by LSD test). a = compared with CsA, b = compared with each corresponding dose of gallic acid alone.

Glutathione peroxidase activity in hearts exposed to I/R was lower than sham group (0.0006 ± 0.0001 vs. 0.001 ± 0.0001 U/mg protein). Significant increase of GPX activity was noticed in G15 mg/kg, G7.5, G15 mg/kg + CsA compared with control (0.0018 ± 0.0002, 0.0015 ± 0.0003, 0.0012 ± 0.0001 U/mg protein, P < 0.01), (Figure 4). In case of gallic acid (15 mg/kg), significant changes in the level of GPX activity were noticed compared with CsA alone (P < 0.01). In addition, a significant elevation in GPX activity was observed in combination of gallic acid 7.5 mg/kg + CsA compared to the same dose of gallic acid alone (P = 0.001).

**Figure 5.**
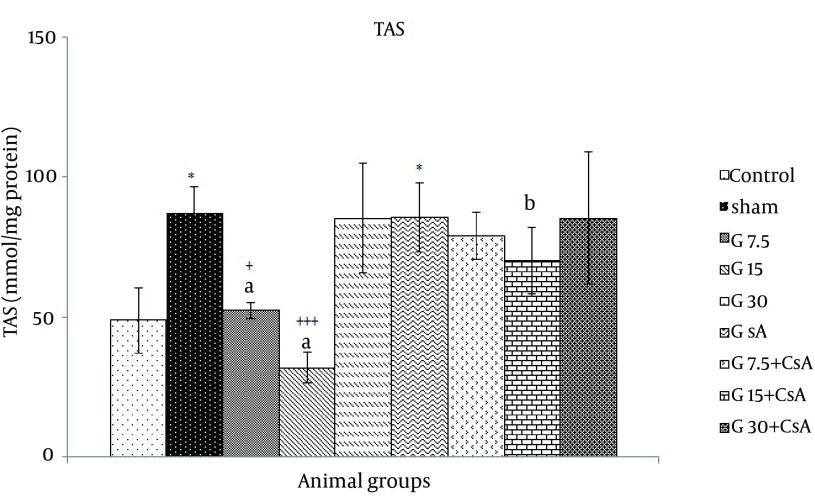
TAS Activity in Heart of Rats After 30 Minutes Ischemia and 60 Minutes of Reperfusion (Mean ± SEM, n = at least 8) The animals received three different doses of gallic acid (G7.5, G15 and G30 mg/kg) for 10 days before the beginning of I/R studies. Animals received cyclosporine A (0.2 µM) at the onset of reperfusion (CsA), Control animals received normal saline as the solvent of gallic acid. Sham animals were not exposed to I/R.* indicates significant difference with control. + indicates significant difference with sham. (P < 0.05, ++ P < 0.01, +++ P < 0.001, one-way ANOVA followed by LSD test). a = compared with CsA, b = compared with each corresponding dose of gallic acid alone.

Total antioxidant status (TAS) was reduced significantly in heart tissue exposed to I/R and pretreated with gallic acid (7.5, 15 mg/kg) compared with sham (49.0 ± 11.5, 52 ± 3, 32.3 ± 6 vs. 61 ± 10 mmol/mg protein, P < 0.05). A significant reduction was observed in TAS activity in groups under gallic acid (15 mg/kg) and G15 + CsA (32.3 ± 6 vs. 70.3 ± 12 mmol/mg protein). Groups that received gallic acid (7.5, 15 mg/kg) compared to CsA alone showed significant reduction in TAS (P = 0.001) (Figure 5).

## 5. Discussion

As expected, our data showed that cardiac marker enzymes and TBARS level increased, but the activity of antioxidant enzymes decreased significantly during I/R. According to our results, pretreatment of animals with gallic acid as an antioxidant increased SOD, CAT and GPX activity; also, reduced cardiac marker enzymes significantly. In addition, lipid peroxidation decreased significantly in the hearts pretreated with gallic acid. Furthermore, reperfusion of hearts by CsA (0.2 µM) for 20 minutes did not decrease cardiac markers significantly, but induced a significant decrease in MDA level and SOD activity, while GPX and CAT activity increased significantly. On the other hand, combination of both drugs had more beneficial effects than each one alone and this effect was significant in CPK and TBARs level. In normal conditions, there is a balance between the formation of prooxidants and amount of antioxidants. During I/R, impairment of myocardial function is mainly attributed to the interruption of the above mentioned balance and elevation of ROS productions (21). As mentioned in introduction, ROS leads to lipid peroxidation of cell and organelle membranes. It worsens the cell depletion from high-energy phosphate due to mitochondrial enzymes dysfunction. Following these events, mitochondrial membrane is depolarized, so the mPTP is opened and electron transformation chain (ETC) disrupts. Then due to anaerobic metabolism and uncoupling of oxidative phosphorylation, mitochondrial ROS production increases and exacerbates the oxidative stress. These perturbations can be restored by inducing SOD and CAT (2, 4, 6). In recent years, it has been recommended that using fresh fruits and vegetables can prevent cardiovascular disease (CVD) due to having natural antioxidants such as flavonoids, anthocyanin and phenolic compounds (9, 22). Antioxidant defense system including scavenging enzymes such as CAT, SOD, GPX, GRX and GST play a critical role in protection of myocardium against oxidative stress. These enzymes decreased and lipid peroxidation increased due to ROS production. Following cell membrane structural damage, the membrane permeability increased and some of intracellular enzymes like Ck-MB, CPK, and LDH leak from the cell, therefore we measured their activity as famous markers of myocardial injury in the coronary effluent (6, 23, 24). In addition, it has been demonstrated that SOD activity reduction leads to accumulation of superoxide, which inactivates GPX, increases H2O2 which inhibits the SOD and makes a vicious cycle (25, 26). In this study, our strategy was to preserve the cell membrane and mitochondria against ROS induced damage by using an antioxidant and preventing opening of the mPTP by a suitable inhibitor (CsA). It has been shown that lysosomal proteases were lowered in Iso-treated rats, so autophagic digestion of mitochondrial proteins occurred because of lysosomal membrane stability reduction (27). In the other hand, other studies showed that gallic acid scavenges directly superoxide and hydroxyl radicals, so prevents the cell membrane against lipid peroxidation. This event preserves the lysosomal membrane integrity due to its antilipoperoxidative and antioxidative effects (14, 28). Furthermore, according to another study, using CsA could preserve mitochondria against oxidative stress by inhibiting the mPTPs during I/R (29). In this study, more reduction of cardiac marker enzymes by combined administration of gallic acid and CsA can be attributed to antioxidative effect of gallic acid as was shown that CAT, SOD and GPX activity increased in hearts pretreated by gallic acid (14). Therefore, the reduction in ROS production probably due to restoration of mitochondrial function could result in SOD and CAT elevation, and then prevention of cell membrane lipid peroxidation and enzyme release. From another point of view, the mitochondrial preservation by CsA could result in less production of ROS. Nevertheless, the two effects of both drugs may partially potentiate each other, as we observed 30% and 50% reduction in CPK by CsA and gallic acid alone respectively, but this effect increased to 70% by their combination, which was more than each one alone. In contrast to other studies, in this experiment, using CsA at the onset of reperfusion showed that GPX and CAT activity increased but TBARS level and SOD activity decreased. These discrepancies can be explained according to the experiment showing that CsA increased ROS synthesis dose-dependently in bovine cultured aortic endothelial cell, but this effect was detectable at a dose of 1 µM for 60 minutes (30). Therefore, increase of antioxidative activity observed in this study can be related to lower dose and duration of using CsA. It seems that CsA in this dose plays a protective role especially because of lipid peroxidation reduction using this dose of CsA. Furthermore, other studies have shown that α–tocopherol, as an antioxidant increased the CAT activity and NADPH bioavailability (31, 32). NADPH was bounded with CAT, and the enzyme inactivated (6, 33). During ischemia, lack of NADPH supply leads to glutathione reductase decrement, so the oxidized glutathione (GSSG) is accumulated and recovery delayed. In this study, combination of gallic acid and CsA partially could normalize the antioxidant capacity. If we suppose that CsA can preserve the mitochondria against oxidative stress, then the mediators of metabolic pathway like NADPH may be maintained and ROS production decreased. Thereafter, the antioxidant enzyme activities can be restored, as we observed in our results, these levels reached to sham group. In addition, TAS as an index of enzymatic and non-enzymatic antioxidants, similar to enzymatic activity was restored by combination therapy. This effect is more obvious in gallic acid 15 and 30 mg/kg + CsA.

In conclusion, the data indicated that pretreatment with gallic acid combined with CsA administration, had more improving effect on antioxidant capacity, cardiac marker enzymes, cell membrane integrity and mitochondrial preservation against oxidative stress due to I/R injury than each one alone.
